# Small fiber neuropathy in hypermobile Ehlers Danlos syndrome/hypermobility spectrum disorder

**DOI:** 10.1111/joim.13539

**Published:** 2022-07-15

**Authors:** Aurore Fernandez, Bérengère Aubry‐Rozier, Mathieu Vautey, Chantal Berna, Marc R. Suter

**Affiliations:** ^1^ Faculty of Biology and Medicine University of Lausanne Lausanne Switzerland; ^2^ Pain Center Department of Anesthesiology Lausanne University Hospital (CHUV) Lausanne Switzerland; ^3^ Center for Integrative and Complementary Medicine Department of Anesthesiology Lausanne University Hospital (CHUV) Lausanne Switzerland; ^4^ Department of Rheumatology Lausanne University Hospital (CHUV) Lausanne Switzerland

Dear Editor,

Hypermobile Ehlers–Danlos syndrome (hEDS) and hypermobility spectrum disorders (HSD) are incapacitating and painful syndromes involving a generalized connective tissue disorder with joint hypermobility and musculoskeletal complications. A neuropathic component seemed clinically likely given frequent burning sensations, hypoesthesia, or allodynia [1]. A small fiber neuropathy (SFN), that is, the dysfunction of A‐δ and C‐fibers relaying thermal, nociceptive, and autonomic information, have been suggested in previous case series of hEDS/HSD [2, 3]. These reports provided early findings of small fiber involvement in hEDS, but their limited sample size (*n* < 20) and sole reliance on skin biopsies and questionnaires called for further investigations.

In our retrospective study, we report structural and functional evaluations of small nerve fibers in a large cohort of patients suffering from hEDS/HSD (*n* = 79, 71% hEDS). Patients were referred to the Pain Center of Lausanne University Hospital when a small fiber neuropathy was suspected based on anamnestic complaints (hypoesthesia, burning pain, allodynia, or dysautonomia). The standardized assessment included a clinical examination, questionnaires, quantitative sensory testing (QST, according to the protocol of the German Research Network on Neuropathic Pain) and skin biopsies (declined by 10 patients) (see Supplementary Material).

We observed a small nerve fiber dysfunction based on QST in 55 of 79 patients (70%) as well as a structural deficit based on intraepidermal nerve fiber density (IENFD) in 54 of 69 patients (78%). Considering only the 69 patients who underwent these two assessments (Fig. 1), SFN was definite (both abnormal structure and function) in 40 of 69 patients (58%), possible (either one or the other) in 23 of 69 patients (33%) and excluded (both normal) in only six of 69 patients (9%).

**Fig. 1:**
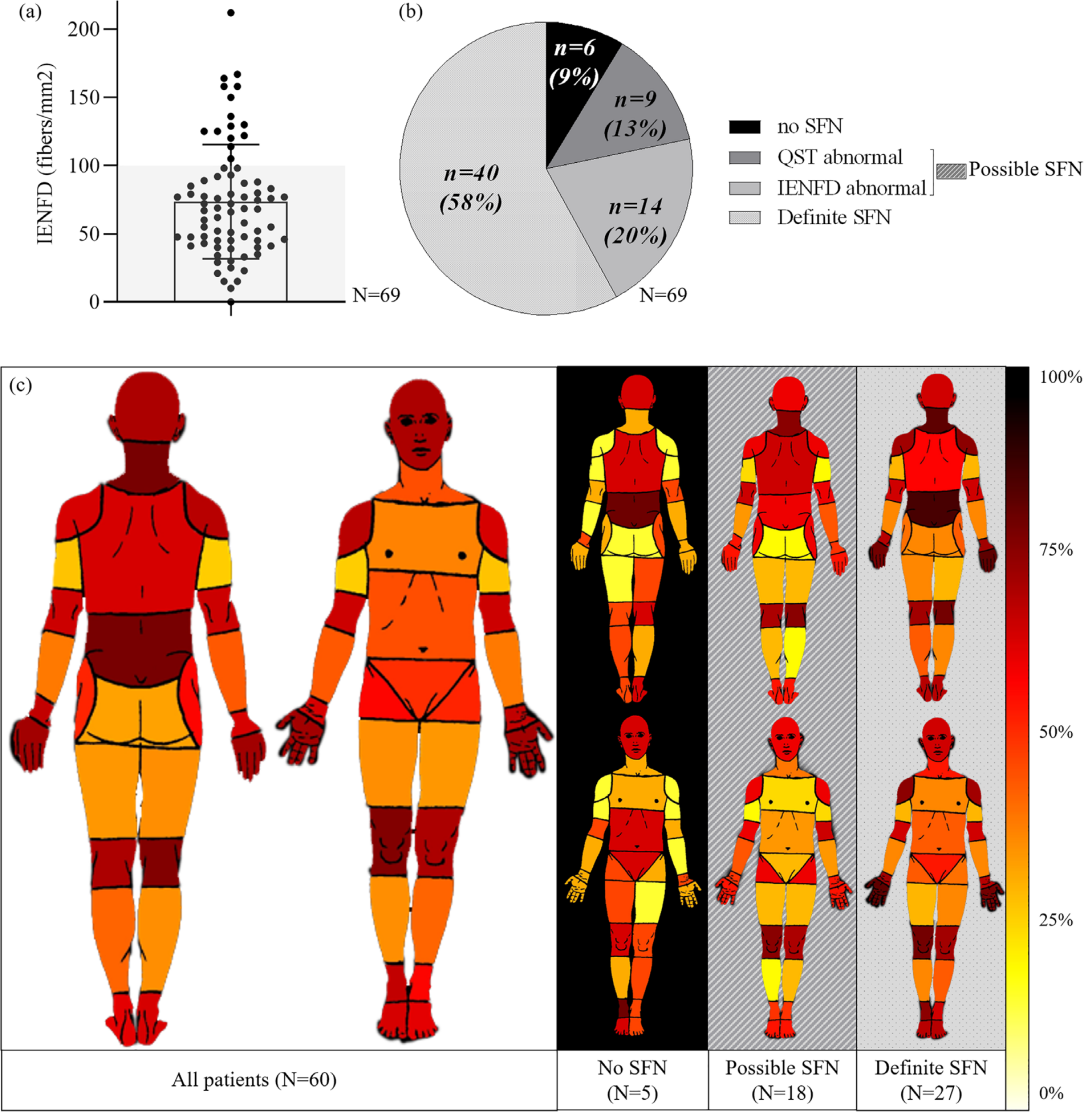
Distribution of the intraepidermal nerve fiber density (IENFD) count in the hypermobile Ehlers–Danlos syndrome (hEDS)/hypermobility spectrum disorders (HSD) population (a); subdivision of the hEDS/HSD population according to the likelihood of small fiber neuropathy based on quantitative sensory testing (QST) and IENFD (b); pain distribution heat map (c). The left part of panel c represents the frequency of pain localization of hEDS/HSD patients as reported on pain drawings; the right panel shows the body map representations in sub‐groups of patients based on their small fiber neuropathy (SFN) likelihood. The color gradient represents the percentage of patients reporting pain in each body area from light yellow (0%) to dark red (100%). Definite SFN = both QST and IENFD abnormal. No SFN = both QST and IENFD normal. Possible SFN = either QST or IENFD abnormal.

Patients in this hEDS/HSD cohort reported at least moderate pain intensity (Brief Pain Inventory [BPI], mean pain intensity, *M* = 5.7, SD = 1.9) interfering with their daily life (BPI–mean pain interference, *M* = 5.7, SD = 2.4). The mean score for the Widespread Pain Index was 11.3/19 (SD = 4.1). Sixty of the 79 patients drew their pain on the BPI body map (with the pain distribution reported in Fig. 1c). On average, 58% of the body zones were considered painful. A sub‐group analysis according to the likelihood of SFN is shown on the right panel of Fig. 1c. The amount of patients reporting pain in the hand significantly differed among these categories (SFN, possible SFN, no SFN; *H*(2) = 8.6, *p* = 0.01), with a significant difference between patients with and without SFN (adjusted *p* = 0.03).

The large proportion of patients in this study displaying small nerve fiber abnormalities underscores the possible contribution of the peripheral nervous system to hEDS/HSD symptoms. Interestingly, unmyelinated small fibers contribute to 80% of joint and musculoskeletal innervation [4]. Afferent nerve fibers also affect muscle development, including spindle formation and maintenance [5]. A loss of sensory innervation impairs the healing of bone fractures. Such a deficit negatively affects tissue development and homeostasis and may contribute to chronic musculoskeletal diseases [6]. SFN could therefore participate to hypermobility through an impairment of development, or an impairement of recovery following repetitive trauma.

Nevertheless, the pain experience in hEDS/HSD is likely to be multifactorial, including a nociceptive contribution (joint instabilities resulting in repetitive dislocations, a muscular component or periarticular inflammation) [7] and a likely central sensitization component [8, 9]. In our cohort, there is evidence for neuropathic, nociceptive musculoskeletal, and nociplastic contributions. The reported proportion of each of these factors varies, which is not surprising given the heterogeneity of the hEDS/HSD population and the various recruitment criteria.

The retrospective design entails some significant limitations. Patients were referred to the pain center because of symptoms compatible with SFN. Hence, the described cohort is not reflecting a full population of hEDS/HSD patients (as described in [10]), but only a significant proportion (50%). The assessment of an entire cohort would allow determining more precisely the SFN prevalence in a general population of hEDS/HSD, including those who are asymptomatic.

The presence of small fiber pathology could help a better stratification of the heterogeneous hEDS/HSD population in terms of severity, disease extent and therapeutic options. Furthermore, the co‐occurrence with SFN, a neurological disorder that can be diagnosed by objective measures, might improve the recognition of the complex hEDS/HSD pathology.

## Conflict of interest

The authors declare no conflict of interest.

## Author contributions

Aurore Fernandez: Conceptualization, data curation, formal analysis, methodology, writing–original draft, and writing–review and editing. Mathieu Vautey: Resources, software, and visualization. Berengère Aubry‐Rozier: Conceptualization. Chantal Berna: Conceptualization, writing‐original draft, writing‐review and editing. Marc Suter: Conceptualization, writing‐original draft, writing‐review and editing.

## Supporting information

Supporting Material
