# Supplementary material for: Small fiber neuropathy in hypermobile Ehlers Danlos syndrome/hypermobility spectrum disorder
Source: J Intern Med. 2022 Jul 15;292(6):957–60. doi: 10.1111/joim.13539 (PMC9796626; doi:10.1111/joim.13539)
Supplement: Supplementary file 1 — Supporting Material [file JOIM-292-957-s001.docx]

Supplementary material: Small fiber neuropathy in hypermobile Ehlers Danlos Syndrome / Hypermobility Spectrum Disorder

Aurore Fernandez^1,2,3^, Bérengère Aubry-Rozier^1,4^, Mathieu Vautey^3^,Chantal Berna*^1,2,3^ and Marc R. Suter*^1,2^

***Detailed Methods***

***Subjects***

This retrospective chart analysis was approved by the Ethics Committee, Vaud, Switzerland (CERVD 2019-00093). The study population consisted of adult patients suffering from hEDS/HSD, followed at the Department of Rheumatology, Lausanne University Hospital, who had signed general informed consent allowing further use of their clinical data. The following three criteria were required for a hEDS diagnosis [1]: (1) Generalized Joint Hypermobility, based on the Beighton score; (2) presence of ≥ 2 of 3 following characteristics: A. ≥ 5 systemic manifestations; B. positive family history; C. musculoskeletal involvement; (3) exclusion of other diagnoses explaining the symptoms. Patients with symptomatic joint hypermobility, yet not fitting these criteria, were diagnosed as Hypermobility Spectrum Disorder (HSD) [2]. Since 2017, hypermobile patients were referred to the Pain Center of Lausanne University Hospital if there was a suspicion of small fiber neuropathy based on anamnestic complaints (hypoesthesia, burning pain, allodynia, dysesthesia or dysautonomia) (supplementary figure 1). Patients were evaluated by a pain physician, filled standardized questionnaires, and underwent QST. Skin biopsies became available from 2020 on and were offered to complete the assessment (hence up to 3 years apart from the initial clinical evaluation). In patients with a suspicion of SFN, an electroneuromyogram (ENMG) was suggested to be performed by neurologists to exclude large fiber involvement.

An average age and gender-matched healthy control group of N=23 was recruited prospectively (CERVD 2020-02259). Exclusion criteria were: neurological symptoms and possible causes of neuropathy such as diabetes mellitus, thyroid dysfunction, hypovitaminosis B12, acute infection, nerve entrapment, HIV or cancer. Participants underwent QST and answered questionnaires.

**Supplementary figure 1. Flowchart illustrating the clinical path and the inclusion of study subjects.** hEDS=hypermobile Ehlers Danlos Syndrome. HSD=Hypermobility Spectrum Disorder. QST=Quantitative Sensory Testing


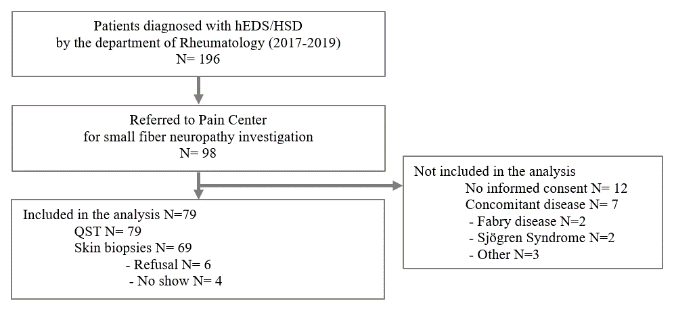


***Symptoms assessment: pain, rheumatological and psychological comorbidities***

Pain intensity and interference with daily life were assessed with the Brief Pain Inventory (BPI) [3]. Pain localization was assessed using the paper body map of the BPI and neuropathic characteristics evaluated with the Douleur Neuropathique 4 questionnaire (DN4) [4]. The widespread pain index (WPI) was used to quantify the pain distribution and the symptom severity scale (SSS) to evaluate symptoms frequently associated with widespread pain such as fatigue, unrestful sleep and cognitive symptoms [5]. We evaluated the psychological health using the Hospital Anxiety and Depression Scale (HADS) [6], fear of movement through the TAMPA scale of kinesiophobia (TSK) [7], and the Pain Catastrophizing Scale (PCS) [8]. Quality of life was assessed through the World Health Organization Quality of Life BREF questionnaire (WHO-QOL BREF) [9]. Raw WHO-QOL-BREF scores were converted into the transformed domains scores ranging from 0 to 100 for physical health, psychological domain, social relationships and environment [9]. The Small Fiber Neuropathy and Symptoms Inventory Questionnaire (SFN-SIQ) was collected [10]. The 13‐items of this self-assessment tool investigate the two clinical presentations of small fiber neuropathy, i.e. autonomic symptoms (changes in sweating pattern, diarrhea, constipation, urinary tract problems like hesitancy and incontinence, dry eyes, dry mouth, orthostatism, palpitations, hot flushes) and sensory symptoms (sensitive leg skin, burning feet, sheet intolerance, and nocturnal restless legs). Each item is scored on a 4‐point Likert scale (0-never present; 1-sometimes, 2-often, and 3-always present). The SFN-SIQ validated cut-off for this scale. The symptom severity of articular and extra-articular manifestations was assessed by a rheumatologist specialized in hEDS/HSD patients, using the Clinical Somatosensory Scale (CSS-16), a 16 items questionnaire assessing pain, fatigue, sleep score is the sum of each item. There is no formally disturbance, mobility, skin, dysautonomia, cardiac, bleeding, gastrointestinal, bladder, temporomandibular joint, ear-nose-throat, lung tract, sexual and cognitive symptoms. Each item was scored on a Likert scale of severity from 0 (absent) to 4 (severe) [11]. The number of participants who answered each of the questionnaires is reported in Supplementary Table 1.

The healthy controls filled the SFN-SIQ, the WPI and the SS. The other questionnaires were not collected, as they have clear clinical cut-offs or data from large reference populations.

***Pain body map analysis***

Patients’ paper body maps from the BPI were scanned; the numerical images were processed using a custom-made program coded in Python, inspired by prior work [12]. Each pain drawing was superimposed to a template divided into 56 body segments. The merged image (drawing + segmentation) was evaluated by two researchers (A.F., M.V) blinded to the identity of the patient. They classified as active each body segment that was filled in some way (i.e. colored, containing a cross, or circled). This allowed to determine the pattern of active segments for each patient. Then, the percentage of patients that reported pain in each body area was calculated and represented on a heat-map [13].

***Quantitative sensory testing***

Quantitative sensory testing (QST) was performed following the standardized protocol of the German Research Network Neuropathic Pain (DFNS) [14] by one experimenter (A.F.). After a log transformation of the raw data to follow a normal distribution, a z-score sensory profile was calculated using the formula: z-score=(value of the patient – mean value of published controls)/standard deviation of published controls). Negative z-scores indicate loss of function and positive z-scores indicate a gain of function. For individual clinical assessment, each patient’s QST values were compared with the corresponding age and gender reference values from the literature [15]. In addition to individual assessments, group comparisons were performed between hEDS/HSD patients and an age- and gender- matched control group, locally recruited and tested in the same experimental conditions. This internal control was added to ensure the observed differences from the published controls could be attributed to patients’ specificities and not differences in experimental setting.

The QST parameters were acquired in the following order: cold detection threshold (CDT), warm detection threshold (WDT), ability to detect temperature changes (thermal sensory limen, TSL), the number of paradoxical heat sensations during TSL (PHS), cold pain threshold (CPT), heat pain threshold (HPT) assessed using TSA II Peltier thermode (Medoc, Israel). Mechanical detection threshold (MDT) was assessed using Von Frey filaments, mechanical pain threshold (pin/prick thresholds, MPT) and mechanical pain sensitivity (MPS) were assessed using pinpricks, dynamic mechanical allodynia (DMA) using a brush, cotton wool and a Q-tip, wind-up ratio represents the pain summation to repetitive pinprick stimuli (WUR), pressure pain threshold (PPT) was assessed using a calibrated algometer (Wagner Instruments, USA), and vibration detection threshold (VDT) using a tuning fork.The testing was performed on a hand and a foot, choosing the anamnestically more affected side except for 3 patients who were tested either on both hands (N=2) or both feet (N=1). For these 3 patients, the data of the most affected limb were considered for analysis. Thermal modalities were not assessed in 1 patient who could not tolerate the thermode due to reported electrosensitivity. CPT, HPT and MPS were not assessed in 1 other patient due to allodynia/hyperalgesia. Missing data were not replaced and the N for each modality is reported on supplementary figure 2.

***Skin biopsies***

Samples were collected at the Lausanne University Pain center and analyzed at Bern University Hospital (Inselspital) as part of routine clinical care. A skin punch biopsy (3mm; device by Stiefel, Germany) was performed under local anesthesia with subcutaneous lidocaine and a topical cold spray (Sintetica, Switzerland) to ensure comfort despite self-reported local anesthetic resistance in many patients. The skin sample was fixed in ready-to-use 4% paraformaldehyde (Biosystems, Switzerland) at 4°C for 1h and stored into a freshly prepared phosphate buffer until processed. The intraepidermal nerve fiber density count took place at the Neuromorphological laboratory of Bern University Hospital (Inselspital). As part of their standard procedure, 50μm cryosections were immunoreacted with antibodies against protein-gene product 9.5 (PGP9.5). PGP9.5-positive nerves were quantified visually using fluorescent microscope. The Inselspital-University of Bern Pathology institute method is based on [16]’s work and a count of IENFD >100/mm^2^ is considered normal.

***Small fiber neuropathy definition***

Although there is not yet a gold standard for the diagnosis of SFN, based on recent recommendations [17, 18], in this cohort of patients with compatible symptoms, we considered there was a SFN if both the structure (=reduced IENFD) and the function (=decreased thermal detection at the hand or the foot on QST) were altered compared to normative values. SFN was excluded if both IENFD and QST were normal. Possible SFN was defined as only one abnormal test (IENFD or QST).

***Statistical analysis***

All the analyses were performed using SPSS Statistics 27 (IBM, Germany). Demographic parameters were compared using unpaired t-tests. For individual patients’ QST analysis, Z scores above 1.96 (gain of function) or below -1.96 (loss of function) were considered as abnormal based on DFNS standards [15]. T-tests were performed to compare normally distributed z-scores between patients and the local control group for each modality. T-tests were used to perform group comparisons (hEDS vs HSD; patients vs controls, SFN vs no SFN). Bonferroni correction for multiple comparisons was applied with a reporting of adjusted p values. A Mann Whitney test was used to compare the number of patients reporting pain in different body areas (Body map data) between diagnostic categories (SFN, possible SFN, and no SFN). Pearson correlations were calculated between pain intensity, IENFD, Z-scores of QST thermal modalities, pain intensity (BPI) and CSS16.


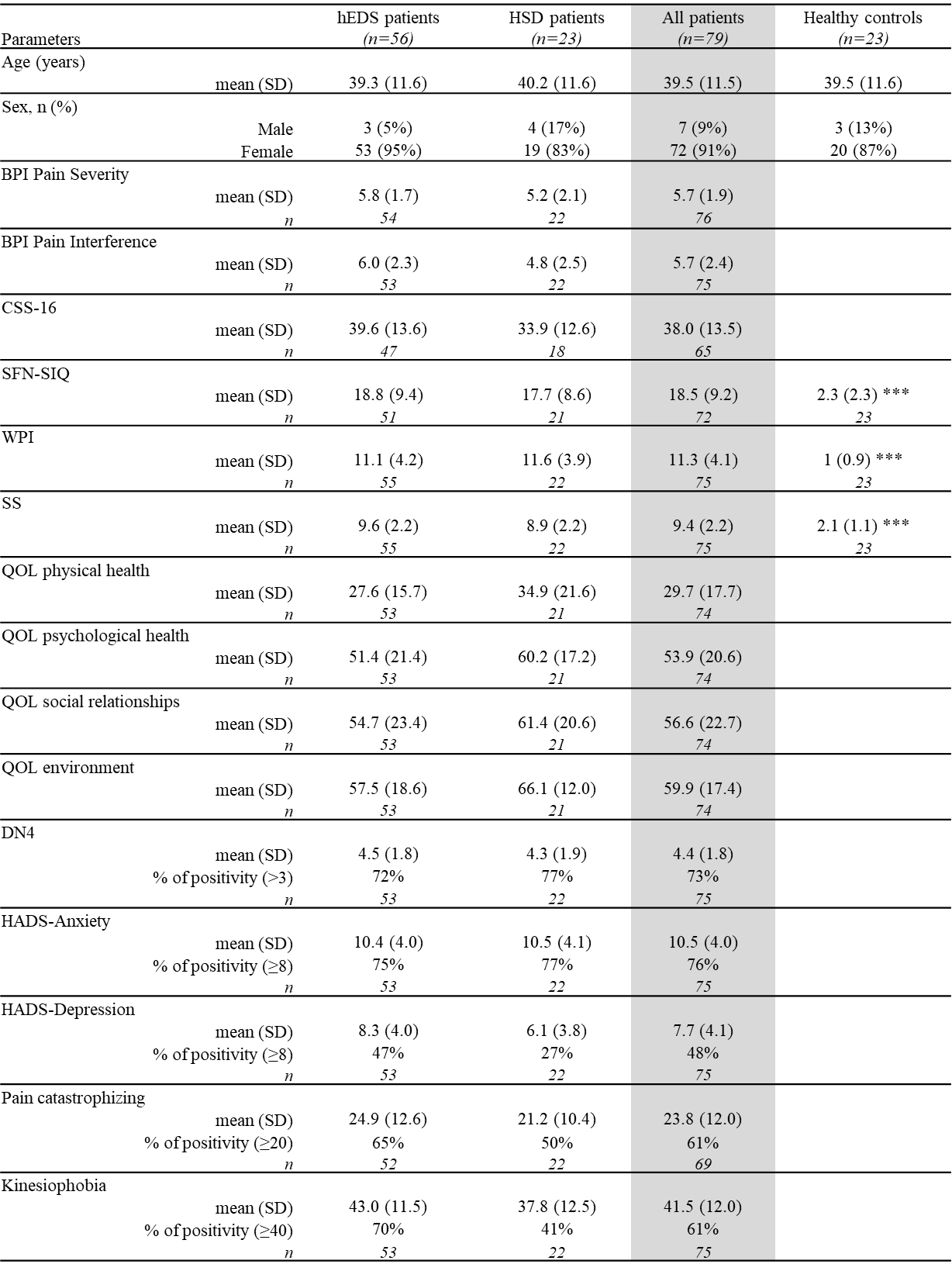


**Supplementary Table 1. Demographic characteristics of the study population and symptoms description.** Mean scores, standard deviation and percentage of positivity=N reaching clinically validated cut-off of scores (considered cut-off) are detailed for patients diagnosed with hypermobile Ehlers Danlos Syndrome (hEDS, first column), Hypermobility Spectrum Disorder (HSD, second column), for all the patients combined (hEDS and HSD, third column in grey), and for age and gender matched local healthy controls (last column). BPI=Brief Pain Inventory; CSS16=Clinical Somatosensory Scale; SFN-SIQ=Small Fiber Neuropathy Symptoms Inventory Questionnaire; WPI=Widespread Pain Index; SS=Symptom Severity; QOL=Quality of Life from WHO-Bref; DN4=Douleur Neuropathique 4; HADS=Hospital Anxiety and Depression Scale; ***= p<0.001, differences between all patients and healthy controls. There were no significant differences between hEDS and HDS for these scores.

***Detailed Results***

***Demographic data and symptoms description***

Demographic data for all the patients are detailed in Supplemental Table 1. Of the 79 patients included in the analyses, 56 fulfilled hEDS diagnostic criteria (71%). The 23 HSD patients failed hEDS criteria because of missing a positive family history (N=10), systemic symptoms (N=3) or both (N=10). There were no significant differences in any of the symptom scales (CSS, BPI, WPI, SS, SFN-SIQ, DN4), quality of life nor psychological health between hEDS and HSD patients. All the patients reported pain (BPI-PS M=5.7, SD=1.9) interfering with their daily life (BPI-PI M=5.7, SD=2.4). The mean score for Widespread Pain Index was 11.3/19 (SD=4.1). Sixty of the 79 patients drew their pain on the body map, with the pain distribution reported in figure 1C. On average, 58% of the body zones were considered as painful. Anxiety (76%), depression (48%), catastrophizing (61%) and kinesiophobia (61%) were frequently observed (supplemental Table 1).

***Quantitative sensory testing (QST)***

Individual sensory profiles are represented in supplementary figure 2. There were no significant differences in sensory profiles between patients diagnosed with hEDS and HSD, justifying the aggregation of the two diagnostic groups. Sensory loss of function of small fibers (i.e., any decrease in thermal detection: CDT, WDT or TSL on either hand or foot) was observed in 55/79 hEDS/HSD patients (70%) when compared to published normative data [15]. Specifically, more than one third of the patients displayed cold and/or for warm hypoesthesia (tables of suppl. figure 2 A and B). Patients also presented hypodetection of temperature changes (TSL, hand: 46%; foot: 37%), hyperalgesia to muscle pressure (PPT, hand: 32%; foot: 14%) as well as hypoesthesia for light touch (MDT, hand: 42%; foot: 32%).

When compared to the local matched controls, patients had significantly increased detection thresholds for cold, warm and temperature changes on both the hand and the foot (see suppl. figure 2, panel C and D). These alterations are compatible with functional impairment of small fibers. In addition, patients displayed an increased wind-up ratio (WUR) as well as a decreased detection of light touch (MDT). Paradoxical heat sensations were observed in 6 patients for the hand (8%) and in 34 patients for the foot (45%) whereas none of the local controls experienced such sensation on the hand, and only two on the foot (9%). Dynamic mechanical allodynia was measured in 9 patients (12%) for the hand and in 11 patients for the foot (14%) but not in controls.

***Intraepidermal Nerve Fiber Density (IENFD)***

There was no difference in IENFD between hEDS (M=77.4, SD=42.5) and HSD (M=62.4, SD=45.5), t(67)=0.8 p=0.4, justifying also a joint analysis. Small nerve fiber density was assessed in 69 of the 79 patients (48/56 hEDS and 21/23 HSD patients). IENFD ranged from 0 to 212 small nerve fibers by mm^2^ (M=74.7, SD=43.3). A reduced IENFD was described in 54 patients (78.2%) (figure 1, panel A).

***Correlations between IENFD and QST***

There was no correlation between IENFD and Z-scores of thermal QST modalities. Furthermore, no correlation was shown between these small fiber integrity outcomes and symptom severity questionnaires (pain intensity and interference, CSS16, SFN-SIQ).

***Small fiber neuropathy (SFN)***

SFN defined as a decrease of both IENFD and thermal detection, was confirmed in 40/69 patients (58%). Possible SFN was detected in 23/69 patients. SFN was excluded in 6 patients (figure 1 B). Among the 10 patients who did not undergo skin biopsy for IENFD count, 6 displayed functional abnormalities (QST).

Fifty out of the 79 patients (63%) underwent an ENMG, which was normal in 40 (80%). As a reminder, this procedure was not performed at our center, and hence, some patients did not comply with the recommendations to consult a neurologist. Among the 10 abnormal ENMGs, 7 revealed a carpal tunnel syndrome, 1 a tarsal tunnel syndrome, 1 a meralgia paresthetica and 1 a distal polyneuropathy. The patient with a polyneuropathy was kept in the SFN group for analysis according to the chosen definition based on structural and functional criteria.

**Supplemental Figure 2. Quantitative Sensory Testing profile assessed on the most affected hand (A and C) and foot (B and D).** Patients’ individual results are presented on the upper panels (A, B). Z-score beyond 1.96 (in the yellow band) correspond to a sensory gain of function and below -1.96 (in the blue band) to a loss of function, in comparison to published normative data. The N and the distribution between gain, loss or normal sensory function are presented for each modality in the tables below panels A and B. Group comparison between patients and the local matched sample of healthy controls are presented in the lower panels (C, D). The tables below panels C and D detail the mean Z-scores and standard deviations for each modality and the statistics: t and p-values, adjusted for multiple comparisons. Statistically significant differences are highlighted in grey. CDT=cold detection threshold. WDT=warm detection threshold. TSL=thermal sensory limen. CPT=cold pain threshold. HPT=heat pain threshold. PPT=pressure pain threshold. MPT=mechanical pain threshold. MPS=mechanical pain sensitivity. WUR=wind-up ratio. MDT=mechanical detection threshold. VDT=vibration detection threshold. *** p<0.001, **p<0.01, *p<0.05.


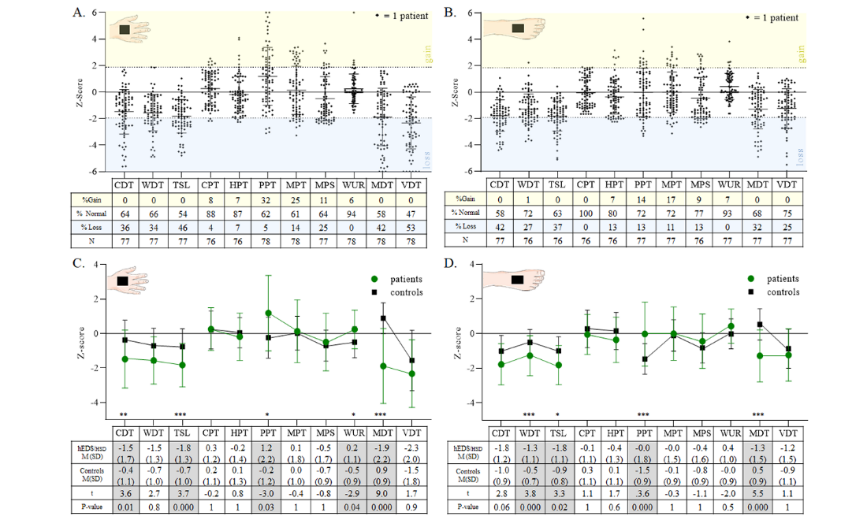


***Differences between patients with and without SFN***

The distribution of the pain localization for the hEDS/HSD population based on the body maps (filled by 60/79) is presented in figure 1C (left panel). A sub-group analysis according to the likelihood of SFN is shown on the right panel of figure 1C. The amount of patients reporting pain in the hand significantly differed among these categories (SFN, possible SFN, no SFN; H(2)=8.6, p=0.01), with a post hoc Bonferroni test showing a significant difference between patients with and without SFN (adjusted p=0.03). Patients with and without SFN showed no significant differences in terms of BPI, CSS16, WPI, SS, psychological scores nor SFN-SIQ.

**Funding**

C.B. was supported by the “Fondation Mercier pour la science” grant. Funding from the University of Lausanne supported the IRB application. The funders had no role in study design, data collection and analysis, decision to publish, or preparation of the manuscript.

**Competing interest**

There are no conflicts of interest.

References

1. Malfait, F., et al., The 2017 international classification of the Ehlers-Danlos syndromes. Am J Med Genet C Semin Med Genet, 2017. 175(1): p. 8-26.

2. Castori, M., et al., A framework for the classification of joint hypermobility and related conditions. Am J Med Genet C Semin Med Genet, 2017. 175(1): p. 148-157.

3. Tan, G., et al., Validation of the Brief Pain Inventory for chronic nonmalignant pain. J Pain, 2004. 5(2): p. 133-7.

4. Bouhassira, D., et al., Comparison of pain syndromes associated with nervous or somatic lesions and development of a new neuropathic pain diagnostic questionnaire (DN4). Pain, 2005. 114(1-2): p. 29-36.

5. Wolfe, F., et al., 2016 Revisions to the 2010/2011 fibromyalgia diagnostic criteria. Semin Arthritis Rheum, 2016. 46(3): p. 319-329.

6. Zigmond, A.S. and R.P. Snaith, The hospital anxiety and depression scale. Acta Psychiatr Scand, 1983. 67(6): p. 361-70.

7. Miller, R.P., S.H. Kori, and D.D. Todd, The Tampa Scale: a Measure of Kinisophobia. 1991. 7(1): p. 51.

8. Sullivan, M.J.L., S.R. Bishop, and J. Pivik, The Pain Catastrophizing Scale: Development and validation. Psychological Assessment, 1995. 7(4): p. 524-532.

9. Development of the World Health Organization WHOQOL-BREF quality of life assessment. The WHOQOL Group. Psychol Med, 1998. 28(3): p. 551-8.

10. Bakkers, M., et al., Small fibers, large impact: quality of life in small-fiber neuropathy. Muscle Nerve, 2014. 49(3): p. 329-36.

11. Aubry-Rozier, B., et al., Are patients with hypermobile Ehlers-Danlos syndrome or hypermobility spectrum disorder so different? Rheumatol Int, 2021. 41(10): p. 1785-1794.

12. Alter, B.J., et al., Hierarchical clustering by patient-reported pain distribution alone identifies distinct chronic pain subgroups differing by pain intensity, quality, and clinical outcomes. PLoS One, 2021. 16(8): p. e0254862.

13. Shaballout, N., et al., From Paper to Digital Applications of the Pain Drawing: Systematic Review of Methodological Milestones. JMIR Mhealth Uhealth, 2019. 7(9): p. e14569.

14. Rolke, R., et al., Quantitative sensory testing in the German Research Network on Neuropathic Pain (DFNS): standardized protocol and reference values. Pain, 2006. 123(3): p. 231-43.

15. Magerl, W., et al., Reference data for quantitative sensory testing (QST): refined stratification for age and a novel method for statistical comparison of group data. Pain, 2010. 151(3): p. 598-605.

16. Koskinen, M., et al., A quantitative method for the assessment of intraepidermal nerve fibers in small-fiber neuropathy. J Neurol, 2005. 252(7): p. 789-94.

17. Devigili, G., et al., Diagnostic criteria for small fibre neuropathy in clinical practice and research. Brain, 2019. 142(12): p. 3728-3736.

18. Egenolf, N., et al., Diagnosing small fiber neuropathy in clinical practice: a deep phenotyping study. Ther Adv Neurol Disord, 2021. 14: p. 17562864211004318.
